# Editorial: Methods in computational genomics

**DOI:** 10.3389/fgene.2024.1367531

**Published:** 2024-01-25

**Authors:** Lei Chen, Nathan D. Olson

**Affiliations:** ^1^ College of Information Engineering, Shanghai Maritime University, Shanghai, China; ^2^ National Institute of Standards and Technology (NIST), Gaithersburg, MD, United States

**Keywords:** genomics, artificial intelligence, machine learning, computational tool, software, bioinformatics

In the rapidly evolving field of Methods in Computational Genomics, this editorial series illuminates the forefront of experimental techniques and methodologies. From dissecting large multidimensional numeric datasets to predicting the functions of novel genomic entities, these approaches have revolutionized our understanding of genomic data. This editorial underscores two pivotal themes: the development of innovative tools and software, and the integration of artificial intelligence (AI) and machine learning in genomic research. These themes exemplify the significant impact computational methods have had on genomics, providing novel insights into complex biological questions.

Theme 1—Tools and Software. Tools and software are fundamental to computational genomics. This section underscores the broader impact of these innovations in advancing genomic research, particularly in metagenomics and gene expression analysis.

Melzer et al. *- CLARITY App*. This application, developed for high-resolution genetic mapping, exemplifies the integration of computational tools with traditional genomics. Its capacity to interconnect physical and genetic maps and visualize recombination hotspots illustrates how software can significantly enhance genomic research, making complex data more accessible and interpretable.

Munro et al. *- Real-time ONT Sequence Analysis Pipeline*. Their tool addresses the need for efficient pathogen monitoring. By optimizing sequencing time and costs, this pipeline demonstrates the practical benefits of computational tools in real-time sequence analysis, which is crucial for epidemiological surveillance.

Liang et al. *–*ARGem*: Antimicrobial Resistant Pipeline.* This user-friendly pipeline for profiling antibiotic resistance genes reflects the growing importance of metagenomics in environmental monitoring. Its high performance in analyzing aquatic metagenomes highlights the tool’s flexibility and utility in diverse research contexts.

Alves et al. *- EasySSR*. This web tool for microsatellite analysis streamlines genomic comparisons, catering to the need for simple yet effective tools in genomic research. EasySSR’s functionality, providing outputs like PTT files and interactive charts, demonstrates how computational tools can facilitate complex genomic analyses.

Each manuscript in this theme showcases how computational tools and software are not just auxiliary but integral to genomic research, offering innovative solutions to traditional challenges and opening new avenues for exploration and discovery in the field.

Theme 2–AI/Machine Learning. This theme highlights the transformative impact of AI and machine learning in computational genomics, showcasing a range of algorithms from statistical learning to advanced deep learning.

Sub-theme 1–Genomic Analysis. Genomic analysis is critical in computational genomics for its ability to unravel complex biological mechanisms.

Ju et al.—*DNA N4-methylcytosine (4mC) Analysis*. The use of deep learning models for predicting 4mC sites, as demonstrated in the brief research report, exemplifies the potential of these advanced techniques in enhancing our understanding of gene regulation and genome stability.

Jia et al.—*Enhancer Prediction*. Jia et al.’s iEnhancer-DCSV method, which employs densely connected convolutional networks, showcases how AI can be leveraged to predict enhancers, thus contributing to the understanding of gene transcription and expression.

Zhang et al.—*CTCF Binding Sites Analysis*. Zhang et al.’s machine learning model for predicting chromatin loop anchors from CTCF binding sites underscores the role of AI in dissecting complex genomic structures.

Deng et al.—*Osteoporosis Genomic Research*. Deng et al.’s investigation into osteoporosis demonstrates the power of bioinformatics in identifying immune-related genetic markers, highlighting the intersection of AI and medical genomics.

Sub-theme 2–Protein/Peptide Analysis. Protein/peptide analysis is essential for understanding complex biological functions and disease mechanisms.

Su et al.—*Outer Membrane Protein Prediction*. Su et al.’s computational model for predicting outer membrane proteins illustrates the application of AI in protein analysis, enhancing our understanding of cellular structures.

Liu et al.—*Neuropeptide Prediction*. Liu et al.’s ensemble tool, integrating multiple convolution neural network models, demonstrates the effectiveness of AI in predicting biologically significant peptides.

Wang et al.—*Antimicrobial Peptide Prediction*. Wang et al.’s deep learning strategy for predicting antimicrobial peptides represents a significant advance in therapeutic research, offering potential applications in treating conditions like diabetic foot.

Livesey et al.- *Cancer Genomic Analysis*. Livesey et al.’s approach to kidney renal clear cell carcinoma employs AI for gene analysis, showcasing how these techniques can lead to meaningful insights in cancer research.

Throughout this theme, the diverse range of AI/ML methods and their specific applications in different genomic contexts highlight the vast potential of these technologies in pushing the frontiers of genomic research.

The transformative impact of these methods is particularly evident in precision medicine. The perspective by Latapiat et al., focusing on individualized co-expression networks, is a testament to this evolution. Their approach to patient stratification in complex diseases underscores the real-world implications of computational genomics, enhancing diagnostics and treatment personalization. This aligns with the ongoing need for tool and software development aimed at optimizing data generation and information extraction, furthering our understanding of biological systems.

This series has showcased remarkable advancements in computational genomics, exhibiting the synergy between innovative software tools, AI, and machine learning techniques. These manuscripts demonstrate how both cutting-edge and established algorithms contribute to the field’s robustness and innovation. From the efficient mapping of genetic landscapes with tools like CLARITY to the sophisticated prediction of neuropeptides using ensemble AI models, these studies exemplify the diverse range of applications in genomics.

As we conclude, the integration of novel computational methodologies with traditional approaches is not just enhancing genomic research but is pivotal in deciphering the complexities of life sciences. The future of genomics, rich with potential, is set to be driven by these innovative computational strategies.

